# Obesity and Cancer: 27-Hydroxycholesterol, the Missing Link

**DOI:** 10.3390/ijms21144822

**Published:** 2020-07-08

**Authors:** Arvand Asghari, Michihisa Umetani

**Affiliations:** 1 Center for Nuclear Receptors and Cell Signaling, Department of Biology and Biochemistry, University of Houston, Houston, TX 77204-5056, USA; aasgharikhonakdari@uh.edu; 2 HEALTH Research Institute, University of Houston, Houston, TX 77204-5056, USA

**Keywords:** 27-hydroxycholesterol, estrogen, estrogen receptor, adipose, obesity, cancer, breast cancer

## Abstract

Obesity is currently affecting more than 40% of the Americans, and if it progresses with this rate, soon one out of two Americans will be obese. Obesity is an important risk factor for several disorders including cardiovascular disease, the first cause of death in the United States. Cancer follows as the second deadliest disease, and a link between obesity and cancer has been suggested. However, it is very hard to establish an exact connection between obesity and cancers due to the multifactorial nature of obesity. Hypercholesterolemia is a comorbidity of obesity and also linked to several cancers. Recently a cholesterol metabolite 27-hydroxycholesterol (27HC) was found to be an endogenous selective estrogen receptor modulator (SERM), which opened new doors toward several interesting studies on the role of this molecule in biological disorders. It is speculated that 27HC might be the missing link in the obesity and cancer chain. Here, we explored the effects of 27-hydroxycholesterol on obesity and cancers with a focus on the SERM capacity of 27HC.

## 1. Introduction

Cancer is the second cause of death after heart disease in the United States, projecting to lead to more than 600,000 deaths in 2020 [1]. According to the recent cancer statistics report, lung and bronchus cancers are leading cancers in an estimated number of deaths in both genders, followed by prostate and breast cancer, in males and females, respectively [1]. In 2019 alone, more than 190,000 new papers have been published and indexed on PubMed on the topic of cancer, ranging from efforts to elucidate the underlying reasons of cancers to advising potential new treatment options. However, there is still a huge gap in our knowledge about cancers, their risk factors, underlying genes and pathways, and how to treat them. Furthermore, obesity is one of the important and established risk factors for several types of cancers, including but not limited to breast and liver cancers [2,3]. However, due to the complexity and multifactorial nature of obesity, it is yet difficult to establish an exact link between cancer and obesity. Some of the proposed mechanisms of action by obesity in the context of cancers are through inducing the inflammation and up-regulating the secretion of inflammatory cytokines, inducing insulin resistance, tumor microenvironment perturbation, hypercholesterolemia, alterations in adipokine pathophysiology, factors deriving from ectopic fat deposition, and increasing the local production of sex hormones biosynthesis, such as estrogen in the adipose tissue [4,5,6].

Estrogen and its classical receptors, estrogen receptor (ER) α and β, are of crucial importance in the development and prognosis of various disorders such as neurodegenerative diseases, bone complications, cardiovascular diseases, cancers, and obesity [7]. ERα and ERβ are differentially expressed in various tissues, and they possess a large ligand-binding pocket which allows different compounds to bind to the receptors [8,9]. While ERα and ERβ are responsible for transcriptional (nuclear) signaling of estrogen, its non-nuclear signaling is also through the binding to a non-classical estrogen receptor, G protein-coupled estrogen receptor (GPER) [10]. Binding of estrogen to GPER triggers multiple downstream pathways that result in the regulation of cell growth, migration, and programmed cell death in a variety of tissues, thus, it may play an important role in cancer. Selective estrogen receptor modulators (SERMs) are synthetic non-steroidal compounds that play very important roles in the above-mentioned disorders as they work as ER agonists or antagonists in a tissue-specific manner, and thus enhance or diminish the effects of ER in the diseases listed above. Since the discovery of the first SERM in the early 1960s, numerous synthetic SERM have been generated. Tamoxifen, one of the most popular SERMs, acts as an ER agonist in bone and uterus but also as an ER antagonist in the breast; therefore, it has been an effective adjuvant endocrine therapy for breast cancer for more than 30 years [7]. Recently, a cholesterol metabolite, 27-hydroxycholesterol (27HC), was found to be a SERM, working as an agonist for ER in the breast, while working as an antagonist in tissues such as bone and cardiovascular system [11,12,13]. This is the first molecule generated inside our body to be known as a SERM.

The goal of this review is to summarize the recent findings on the crucial impacts of 27HC on obesity and cancers through its SERM activity on classical estrogen receptors. As ERs regulate various disorders and 27HC is the first and only endogenous SERM up to now, exploring the roles of this major cholesterol metabolite in the etiology of diseases is of paramount importance.

## 2. 27-Hydroxycholesterol, an Endogenous Selective Estrogen Receptor Modulator

**Figure 1:**
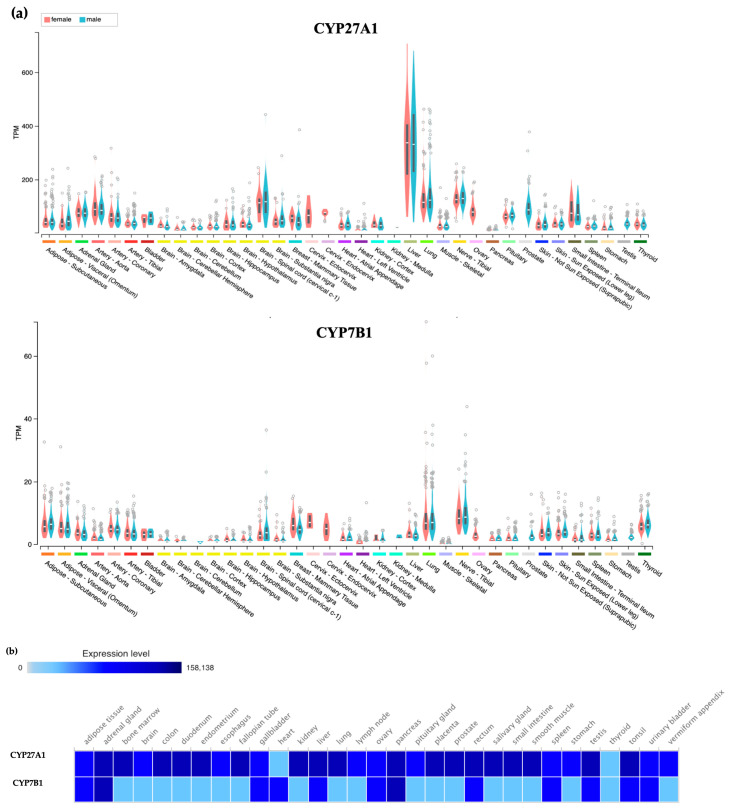
Expression Patterns of CYP27A1 and CYP7B1 in humans. (**a**) The gene expression of *CYP27A1* and *CYP7B1*. The expression data were based on the RNA expression data obtained from more than 1000 individuals. The results were obtained from the GTEx Portal. (**b**) The expression of CYP27A1 and CYP7B1 proteins. The expression data of these enzyme protein levels were obtained from the work of Wang et al. which categorized the proteome data of 29 healthy human tissues [19]. The visualization of the results was done via Expression Atlas [20].

Oxysterols are metabolites of cholesterol that are produced in the liver and other peripheral tissues as a means of excess cholesterol elimination [14]. With serum levels correlated with those of cholesterol, 27HC is the most abundant oxysterol in circulation [15]. In healthy humans, the 27HC level varies between 0.2 to 0.9 μM, while its concentration increases drastically in hypercholesterolemia and also with age [16,17]. It is enzymatically generated from cholesterol by sterol 27-hydroxylase (CYP27A1) and catabolized toward bile acids by oxysterol 7α-hydroxylase (CYP7B1) [14]. It is noteworthy that CYP27A1 resides on the inner membrane of mitochondria and relies on the steroidogenic acute regulatory protein (StAR) for the delivery of cholesterol for 27HC synthesis [18]. Therefore, any disruption in this transportation pathway can impact the 27HC production. While both CYP27A1 and CYP7B1 are mainly expressed in the liver to facilitate the metabolism of excess cholesterol to bile acids, they are also expressed in lung, brain, adipose, and other peripheral tissues. Of note, the expression levels of these enzymes in protein level is somewhat different from their RNA level expression, which needs to be considered when studying them (Figure 1).

In the field of nuclear receptors, 27HC was first characterized as an endogenous liver X receptor (LXR) ligand, increasing the activity of both LXRα and LXRβ in a dose-dependent manner [21]. However, back in 2007, our group demonstrated that 27HC inhibits the activation of ERα and ERβ by 17β-estradiol (E2) in a Gal4-ER ligand-binding domain (LBD) one-hybrid assay in mammalian cells, and also confirmed that this effect is through the direct binding of 27HC to ERα (Ki = 1.32 μM) and ERβ (Ki = 0.42 μM) using in vitro assays [13]. Furthermore, we showed that 27HC is a competitive ER antagonist in the cardiovascular system, where it inhibits the ER-dependent production of nitric oxide (NO) and expression of vascular NO synthase in vascular endothelial cells, leading to decreased vascular healing after injury and increased development of atherosclerotic lesions [13,22]. However, when we explored the potential effects of 27HC on ERs in other cell types, we found that 27HC antagonized ER transactivation in bovine aortic endothelial cells, while it showed pro-estrogenic activity in HepG2 (hepatoma) and Caco-2 (colon cancer) cells. In addition, we found that 27HC changes the conformation of ERα uniquely from E2 binding to ERα, suggesting that 27HC is a selective estrogen receptor modulator, or SERM [13]. Following the identification of 27HC as a SERM, several studies have been published on the impacts of 27HC in the pathologies of various diseases including cardiovascular diseases, osteoporosis, Alzheimer’s disease, obesity, and several cancers [11,12,13,23,24,25,26,27].

## 3. 27HC in Obesity

Obesity, or an increase in general adiposity, is defined as a body mass index (BMI) of more than 30 kg/m^2^ [28]. It is estimated that more than 42% of the US people are obese, while more than half of the Americans are either overweight or obese (BMI ≥ 25 kg/m^2^) [29]. With obesity rates skyrocketing in the United States and globally, the concerns over obesity-related diseases are growing quickly, which stimulates immense research interests in the adipose tissue and its functions. White adipose tissue (WAT) is prominently known as an energy storage site mostly due to its limitless capacity in storing triglycerides [30]. However, this tissue does way more than just as a reservoir for energy storage. Now, WAT is recognized as a very important organ in energy, insulin, and glucose homeostasis, and also as an endocrine organ as well as a major site for the steroid metabolism [31,32,33]. Both classical estrogen receptors are expressed in adipose tissues with ERα being more dominantly expressed [34]. GPER is also expressed in adipose tissues, and while reports of GPER knockout mice show an increase in adiposity and sometimes impaired insulin secretion and glucose intolerance, its importance, and role in adipose tissues is not fully clear yet [10,35,36]. ERα is crucial for protecting against weight gain and maintaining glucose and insulin homeostasis, as the deletion of this receptor in mice (αERKO mice) led to a significant increase in the bodyweight as well as the weight of fat depots, insulin resistance, and impaired glucose tolerance [37]. In the case of ERβ, while systemic ERβ knockout in male mice did not show any changes in the body weight, fat distribution, or the serum leptin and insulin levels [38], removing E2/ERβ signaling in αERKO female mice by ovariectomy decreased body and fat-pad weights and adipocyte size, while improving insulin and glucose metabolism, suggesting that ERβ-mediated effects on adipose tissues oppose those by ERα, and E2 effects on adipose tissues were predominately through ERα [39]. These results show the paramount role of ERα in the regulation of metabolic functions in adipose tissues and obesity, and signify the potential role of ER modulators as regulators of obesity and obesity-related diseases. Tamoxifen blocks ER activities in the breast tissue while it activates ERα in the bone. In regard to adipose tissues, tamoxifen treatment in mice led to a significant reduction in fat mass of the animals, while not changing the overall body weight [40], and also changing the morphology of WAT and transitioning it toward brown adipose tissue in female mice [41].

Since serum 27HC levels closely correlate with cholesterol levels, and hypercholesterolemia is a comorbidity of obesity, altered ER-mediated adipogenesis by the actions of 27HC can be of great importance in obesity [23]. A couple of studies regarding the role of 27HC in adipose tissues reported that 27HC treatment on 3T3-L1 (preadipocyte) cells significantly reduce the intracellular triglycerides by down-regulating lipogenic and adipogenic gene expression [42,43]. These studies were based on cell culture experiments. Using the primary culture of stromal vascular fraction from mouse WAT, we also observed that 27HC did not increase the differentiation to mature adipocytes [27]. Therefore, in cell culture models, 27HC inhibits adipogenic gene expression and acts against adipogenesis. In contrast, we found that 27HC significantly induced adiposity and weight gain in vivo, changed adipose tissue morphology, and also increased inflammation in the tissue regardless of the diet [27]. These results suggest that 27HC impacts not only on adipocytes but also on their local environment. Initially, we explored the body weight changes of ovariectomized female wild-type and CYP7B1 null mice during a high fat/high cholesterol (HFHC) diet feeding and treatments with E2 or vehicle control for 8 weeks. Wild-type mice treated with vehicle predictably had a marked body weight gain, and the effect was diminished by E2, which matches the protective role of E2 against weight gain. However, CYP7B1 null mice, which have elevated 27HC levels in the circulation and tissues, showed significant weight gain in the presence of an HFHC diet, and this was not attenuated by E2. These results suggest that 27HC blocked the protective role of estradiol in adipose tissue [27]. To avoid the effect of systemic CYP7B1 deficiency and also explore the direct effects of 27HC on adiposity, we used wild-type mice and fed them with an HFHC diet or normal chow, and treated them with 27HC or vehicle for 8 weeks. While 27HC did not change the body weight gain in the presence of normal chow compared to vehicle treatment, 27HC significantly increased the body weight gain compared to the vehicle control in the presence of an HFHC diet. Next, by measuring the body composition (total body fat and lean mass) of mice using a small animal MRI system, we showed that the 8-week 27HC treatment caused significantly higher levels of body fat and lower levels of lean mass than their vehicle-treated counterpart regardless of the diet, indicating that 27HC increases body fat mass regardless of its body weight regulation. E2 decreases adiposity and body weights; however, 27HC increases them, indicating that the effect of 27HC is not just inhibition of estrogen actions. By repeating the experiments using αERKO mice or LXR α/β null mice, we confirmed that the increase in adiposity and weight gain upon treatment with 27HC is dependent on ERα and not on LXR, another nuclear receptor family that has oxysterols as its ligands. Using metabolic analyses, we also confirmed that this weight gain is independent of food intake and calorie absorption, and is through the direct effects of 27HC on the adipose tissue. Next, by measuring adipose cell sizes and tissue DNA contents, we showed that the increase in WAT weights is due to hyperplasia and not hypertrophy. To elucidate the mechanism of action by 27HC in WAT, we performed mRNA quantification and RNA-seq analysis, and found that 27HC up-regulated inflammatory-related genes and induced adipose inflammation regardless of the diet, similar to the effects by an HFHC diet alone. This study, to the best of our knowledge, is the first to explore the relation between 27HC and adipose tissues in vivo, and it opened the door toward further exploration of this connection, as the inhibition of 27HC can potentially help in reducing obesity and regulating obesity-related diseases such as cancers [27].

## 4. 27HC and Cancer

The presence of a link between obesity and cancer has been disputable for the several past decades. Currently, it is accepted that obesity is one of the established risk factors of cancers [2,3,44], accounting for more than 14% and 20% of cancer deaths in men and women, respectively [45]. Epidemiologic studies clearly linked obesity to increased risk of cancer of at least 13 anatomic sites including breast, endometrial, pancreatic, and ovarian cancers [6]. Another recent umbrella review of systematic reviews and 204 meta-analyses further confirmed the connection between obesity and cancers of the digestive system and hormone-related cancers in women [46]. However, the exact biological mechanisms that link obesity to cancers are still largely unknown. Hypercholesterolemia is a comorbidity of obesity, and the link between cholesterol and carcinogenesis has been studied for a long time; yet it is full of controversies [25,47]. Several epidemiologic studies reported a positive association between low serum cholesterol levels or the use of statins, cholesterol-lowering drugs, and lowered risk of cancers [48,49,50,51], while others suggest no association [52,53,54], and even that low cholesterol levels and statin use might be carcinogenic [55,56,57]. As mentioned earlier, 27HC functions as one of the main connections between cholesterol and obesity, so it is very plausible that 27HC is the one that links obesity/hypercholesterolemia to carcinogenesis and cancer development. Here, we review the recent findings of the roles of 27HC in cancers.

## 5. 27HC and Breast Cancer

Breast cancer is still the most common type of cancer in women in the United States, accounting for more than 30% of new cases in 2020 [1]. The 5-year relative survival rate of breast cancer is reported to be around 90%, one of the highest survival rates in cancer patients [1]. However, it is still the second cause of death from cancer in women in the US, with more than 42,000 deaths estimated for 2020 [1]. Current treatment options for breast cancer consist of initial surgeries to remove the tumor, followed by various drug treatments based on the cancer sub-type over the years. While ER-positive, PR-positive, and HER2-positive breast cancer tend to have more tailored treatments, in the case of ER-negative or triple-negative (ER-negative, PR-negative, and human epidermal growth factor receptor 2 (HER2)-negative) breast cancer (TNBC), there are almost no targeted therapies available so far, and options are limited to routine chemotherapies [58]. All these emphasize the need to explore breast cancer characteristics and risk factors, in the hope of finding better treatment options. Currently, evidence suggests a paradoxical role for obesity in breast cancer. High BMI associates with a reduced risk of premenopausal breast cancer, whereas it strongly correlates with an increased risk of breast cancer after menopause [59]. A meta-analysis of 34 studies comprising more than 2.5 million women showed that postmenopausal breast cancer risk positively associates with each 5 kg/m^2^ increase in BMI [60]. Moreover, a recent detailed review confirmed the positive correlation of obesity with breast cancer [61]. However, the connection between cholesterol and breast cancer is controversial [62]. A recent large study of over 664,000 women found a negative association between hyperlipidemia and breast cancer. This study showed that women over the age of 40 with high cholesterol levels were 45% less likely to develop breast cancer than those without high cholesterol levels, and of patients who developed breast cancer, those with high cholesterol levels were less likely to die (40%) [63]. In contrast, several other meta-analyses suggest a protective effect of low cholesterol and statins administration in breast cancer prognosis [64,65].

**Figure 2:**
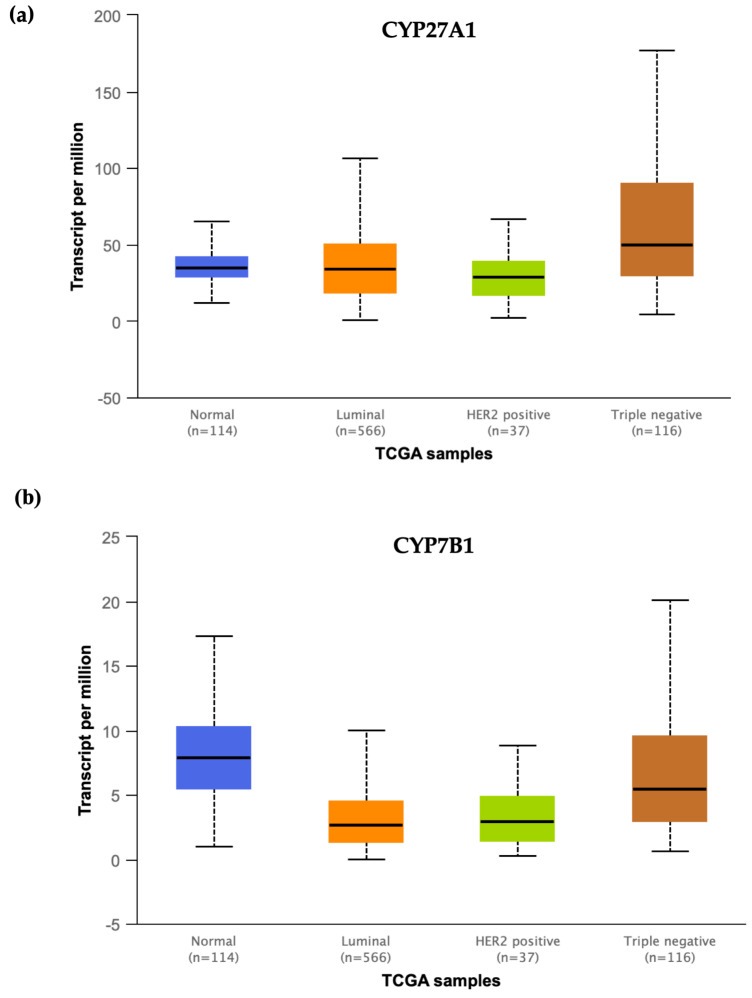
mRNA expression of *CYP27A1* and *CYP7B1* in different subtypes of breast cancer. (**a**) The expression level of *CYP27A1* is significantly upregulated in TNBC compared to normal tissues (*p*-value < 10^−6^), while it is not different in ER-positive or HER2-positive group compared to the normal group (*p*-value > 0.5). (**b**) *CYP7B1* mRNA expression is significantly lower in both luminal (*p*-value < 10^−11^) and HER2-positive (*p*-value < 10^−5^) tumor groups compared to normal breast tissues, while it is not different in TNBC compared to normal tissues (*p*-value > 0.5). The expression data are based on the TCGA data and visualized by the UALCAN portal [69].

Cholesterol-enriched diets are strongly associated with increased risk of breast cancer in mice [25,66,67]. In 2013, two groups reached the same conclusion: 27-hydroxycholesterol links hypercholesterolemia and breast cancer [23,68]. Nelson et al. demonstrated that 27HC increased the breast cancer tumor growth in an ER-dependent manner in an immune-competent mouse mammary tumor virus-polyoma middle T-antigen (MMTV-PyMT) model, which develops spontaneous ERα-positive mammary adenocarcinomas that metastasize to the lung [23]. They also showed that 27HC increased the epithelial to mesenchymal transition marker expressions in a similar manner to the effects of other LXR ligands, which suggests that probably not all of the impacts of 27HC on breast cancer are through ER [23]. The property of cholesterol-enriched diets in the induction of breast cancer tumor growth was lost in the mice lacking the CYP27A1 enzyme, and that statin or a CYP27A1 inhibitor also attenuated the effects of a high-fat diet on the growth of E0771 tumors in transgenic human APOE3 replacement mice, further confirming that 27HC links cholesterol metabolism and breast cancer [23]. Using a human breast cancer MCF7 cell xenograft model into ovariectomized female immunodeficient (SCID) mice, our group found that 27HC administration increased ER-positive breast cancer tumor growth [68]. The intra-tumor concentration of 27HC is around six-fold higher than those of normal breast tissues, and *CYP7B1* gene expression is diminished in the majority of breast cancers, leading to an increase in the abundance of 27HC in breast cancer tumors [68]. Our analysis of the “The Cancer Genome Atlas (TCGA)” data also shows that the expression of *CYP27A1* seems to remain the same in breast tumors compared to normal breast tissues, while the *CYP7B1* expression is significantly lower in tumor samples compared to normal breast tissues, which further confirms the results of our study (Figure 2). Interestingly, in the case of TNBC, *CYP27A1* expression is significantly upregulated in tumor samples compared to normal breast tissues, while *CYP7B1* expression remains the same, suggesting a different regulatory mechanism in TNBC compared to ER-positive breast cancer (Figure 2).

More recently, it was reported that 27HC is also important for the angiogenesis of breast tumors, where 27HC increased the expression of VEGF by the classical ERα/VEGF signaling in ER-positive breast cancer cells [70]. Interestingly, in both ER-positive and ER-negative breast cancer cells, 27HC enhanced the generation of reactive oxygen species, which in turn activates the STAT-3/VEGF signaling in an ER-independent manner [70]. Moreover, it was shown that 27HC facilitated the metastasis of breast cancer by affecting immune cells, mainly by increasing the number of polymorphonuclear-neutrophils and γδ-T cells at distal metastatic sites [71]. While all of these studies suggest a crucial role for 27HC in breast cancer development, growth, and metastasis, there are some controversial clinical results that need to be addressed. Kimbung et al. observed that higher tumor expression of CYP27A1 mRNA in ER-positive breast cancer patients was associated with longer overall survival and recurrence-free survival in ER^+^ breast cancer patients under the age of 50 [72]. However, higher mRNA expression of CYP27A1 does not necessarily reflect the higher protein expression as mentioned earlier and also in the study by Kimbung et al., which makes the interpretation of the results much more complicated. For example, higher levels of CYP27A1 protein is associated with higher tumor grade [23,72]. Moreover, menopause status of the women can play a very significant role in the interpretation of the 27HC relation with breast cancer. In pre-menopause women, significant levels of circulating estrogens are present, so it is plausible that 27HC competes with estrogens for binding to ER, thereby reducing the estrogen-activated ER functions, thus exhibiting its protective role. In contrast, after menopause with a significant loss of endogenous estrogens, 27HC binding to ER stimulates ER transcriptional activity, thus helping in the progression of breast cancer. Women under the age of 50 are mostly in the pre-menopausal stage, and higher levels of 27HC can be expected to be protective against breast cancer, as reported by Kimbung et al. More recently, an epidemiologic study found that high 27HC levels in circulation are associated with a lower risk of breast cancer in postmenopausal women at blood collection [73]. The authors concluded that the “benefit” of the 27HC-associated decrease in the estradiol-ER binding overcomes the “harm” of the partial agonistic effect of 27HC in breast cancer [73]. However, the blood was collected only once for this study and the levels of 27HC might not necessarily reflect the long-term changes in the individuals, which authors themselves mentioned as the limitation of the study. All in all, these clinical studies are the first studies of this kind on 27HC in the clinical settings. While they bring uncertainty about the ultimate role of 27HC in breast cancer, it demands further experiments and studies to elucidate the role and function of 27HC in human breast cancer.

## 6. 27HC and Endocrine Resistance in Breast Cancer

ER-positive breast cancer is the most common subtype of breast cancer among women, and ER-targeted endocrine therapies are the most successful therapies for this subtype of breast cancer [74]. However, despite the initial success of these therapies, tumor recurrence and endocrine therapy-resistance occur in many of the cases. Recently, LBD mutations in the ESR1 gene that encodes ERα were shown to be present in ~18% of endocrine therapy-resistant hormone receptor-positive breast cancers [75,76]. While the discovery of ESR1 mutations offers valuable insights into the evolution of breast tumors under the selective pressure of therapy, only a fraction of breast cancer patients with endocrine-resistant tumors harbors these mutations. In the remainder of patients, the mechanisms of endocrine resistance remain largely unexplained. Recent evidence indicated that 27HC might be one of the underlying factors of endocrine therapy-resistance in patients. When MCF7 cells were deprived of estrogens for a long period—so-called long term estrogen deprivation (LTED), which mimics patients undergoing aromatase inhibitor therapy to block estrogen biosynthesis—a significant upregulation of 27HC biosynthesis occurred by increased expression of CYP27A1 [77]. The same results were observed in patients, where treatment with aromatase inhibitors such as exemestane and letrozole for one month led to the increased levels of circulating 27HC [78]. Moreover, in LTED-MCF7 cells, genes associated with cholesterol homeostasis are significantly upregulated; in contrast, when ER was lost in the cells, cholesterol biosynthesis pathways were not altered [49], indicating the importance of ER in this regulation. It is suggested that 27HC may work as a substitute for E2, bind to ERα, and regulate the downstream pathways; thus it may assist the development of resistance [62]. Indeed, 27HC administration stimulates the growth of tamoxifen-irresponsive MCF7 cells [23]. All these works suggest a potential role of 27HC in endocrine therapy resistance of breast cancer, yet it needs extensive studies before any certain conclusions can be drawn.

## 7. 27HC and Prostate Cancer

Prostate cancer is estimated to have the most number of new cases (more than 190,000 cases) among all types of cancers in male Americans in 2020, while it is the second cause of death from cancer (more than 30,000 cases annually) next to lung and bronchus cancers in males [1]. Due to the multifactorial nature of prostate cancer, the underlying causes of prostate cancer are largely unknown [79]. Epidemiologic studies found several risk factors for this disease, including hypercholesterolemia and obesity [80,81]. The connection between BMI and prostate cancer is controversial as several studies suggested a positive correlation, while others reported an inverse or no correlation, all being reviewed in detail in the literature [82,83]. High plasma cholesterol levels are linked to the increased risk of prostate cancer in multiple epidemiologic studies [50,51,84,85]. However, statins that inhibit cellular cholesterol biosynthesis, thereby lower plasma cholesterol, were unable to improve prognosis in prostate cancer patients, suggesting that cholesterol itself might not be the critical factor in prostate cancer development [86,87]. As a major cholesterol metabolite, 27HC has attracted the attention of prostate cancer investigators over the past decade, and it was demonstrated to stimulate the proliferation of RWPE-1 normal prostate epithelial cells in an ER- and androgen receptor (AR)-dependent manner [88]. Furthermore, 27HC induced AR transcriptional activity and expression of AR-target genes in RWPE-1 cells, and as 27HC does not directly bind to AR, it is possible that there is potential crosstalk between ER and AR in the presence of 27HC in prostate cancer [88]. Later on, the same group showed that 27HC increased the expression of ERβ, but not ERα, and enhanced the growth of prostate cancer cells (LNCaP and PC3) in an ERβ-dependent manner [89]. They also showed that 27HC decreased prostate cancer cell invasion in an ERβ-dependent manner [89]. Interestingly, the CYP27A1 expression level is inversely correlated to prostate cancer development and progression [79], while the CYP7B1 enzyme level is significantly upregulated during prostate cancer prognosis [90], suggesting that 27HC might be actually beneficial against prostate cancer. Despite all the studies on the effects of 27HC on prostate cancer, there are still unanswered questions left, and further studies are needed in order to elucidate the exact role of 27HC in prostate cancer.

## 8. 27HC and Other Cancers

In addition to breast and prostate cancers, 27HC plays a significant role in a variety of cancers. In the case of lung cancer, the most common cause of death from cancer in the US [1], we found that 27HC promotes lung cancer cell proliferation in an ERβ-dependent manner [26]. The expression of *CYP27A1* is higher in lung cancer cells than in normal lung cells. Interestingly, 27HC administration increased cell proliferation in ERβ-positive lung cancer cells, but not in ERα-positive or ER-negative cells. Moreover, using kinase inhibitors, we demonstrated that the effect of 27HC was mediated by the PI3K-Akt signaling pathway [26]. Another group working on endometrial cancer, the most common gynecological malignancy, showed that 27HC contributes to the risk of endometrial cancer by promoting the proliferation of endometrial cancer epithelial cells and also the activation of ER-dependent transcription in well-differentiated endometrial cancer cells [91]. A recent study demonstrated the effects of 27HC on colorectal cancer, the third most diagnosed cancer in the western world [91]. Treating Caco2 and SW620 colon cancer cells with 27HC led to a reduced cellular proliferation. Interestingly, 27HC-induced reduction in cell proliferation was independent of LXR and ER activation. Instead, 27HC significantly decreased the activity of Akt, one of the major protein kinases involved in the pathogenesis of cancer as it regulates cell cycle progression, protein synthesis, and cellular survival [92]. In regard to glioblastoma, 27HC promoted proliferation, epithelial to mesenchymal transition, colony formation, migration, and invasion of U251 and U118 MG glioblastoma cells [93]. Additionally, high 27HC levels in glioblastoma tissues were associated with poor outcome in patients [93]. All these studies showed a potentially critical role of 27HC in many cancers, and further research is warranted to elucidate the exact effects of 27HC in various cancers.

## 9. Enzymes and Drugs that Lower 27HC Levels

As described above, increased levels of 27HC trigger many metabolic dysfunctions and cancers. Therefore, lowering 27HC levels seems effective to ameliorate or prevent such diseases. As described before, CYP7B1 is the catabolic enzyme of 27HC. Both CYP7B1 and the 27HC-producing enzyme CYP27A1 are regulated by various factors such as cytokines and hormones [94]. In addition, hydroxysteroid sulfotransferase decreases the levels of cholesterol and oxysterols including 27HC [95,96]. Although the expression and function of this enzyme are related to some cancers including skin, liver, and gastric cancers [96,97], the enzyme is not specific to 27HC and its physiological function is not yet clear. In regard to pharmacological interventions of 27HC levels, statins, cholesterol-lowering drugs, and some CYP enzyme inhibitors reduce the 27HC levels as well, but they are not specific to 27HC, so it is hard to examine the direct impact of decreased 27HC on the disease [72]. Potential drugs against CYP27A1, a cytochrome P450 enzyme responsible for the conversion of cholesterol to 27HC, can decrease the levels of 27HC in circulation and thus potentially stop the progression of diseases, although systemic CYP27A1 inhibition alters cholesterol and bile acid metabolism, especially in the liver [98]. While some of the current FDA-approved drugs such as cyclosporine and several aromatase inhibitors have shown some inhibitory effects on CYP27A1 [62], more specific therapies against CYP27A1 may be beneficial.

## 10. Conclusions and Future Remarks

In the modern world, the rate of obesity is increasing annually, and dietary habits of individuals make the fight against obesity a very difficult mission. Thus, there is a great need to understand the underlying mechanisms of obesity and how it can contribute to other diseases such as cancers. We speculate that 27-hydroxycholesterol can be the link, at least in part, between obesity, hypercholesterolemia, and cancers. The discovery of 27HC as an endogenous SERM led to extensive studies on the potential roles of this molecule in many diseases including cancers. Initial pre-clinical models suggested an important role for 27HC in atherosclerosis, osteoporosis, and Alzheimer’s disease. Other studies found other significant roles for 27HC in cancers, such as breast, lung, colorectal, brain, and prostate cancers. Interestingly, the identification of the role of 27HC in adipose tissue and obesity shed light on the new potential mechanisms of effects caused by 27HC in cancers. In breast cancer, adipose tissues form a major part of the breast tissue, thereby breast tumors are surrounded by adipose tissues. Since 27HC directly affects WAT and induces inflammation in this tissue, it is plausible that the inflammation in the microenvironment of the breast can contribute to the progression and development of cancer. Moreover, 27HC may be also crucial in several yet-to-be-studied hormone-related cancers, such as uterine, ovarian, and testicle cancers, as obesity and hypercholesterolemia are among the important risk factors of these cancers. While all these studies showed a crucial role for 27HC in these disorders, the results are still controversial and further experiments are warranted to completely elucidate the role of 27HC in cancers. The lack of in vivo and clinical studies is a huge gap in our knowledge of 27HC and its potential roles along various diseases from obesity to cancer, so future clinical studies are extremely needed and crucial in filling out such gaps. As the effects of 27HC in murine models of some of the cancers are elucidated, there is a need to devise drugs and inhibitors to decrease the levels of 27HC both at tumor sites and also the microenvironment of the tumors in the hope of blocking the prognosis of cancers.

## Abbreviations

27HC	27-hydroxycholesterol
SERM	Selective estrogen receptor modulator
ER	Estrogen receptor
GPER	G protein-coupled estrogen receptor
CYP27A1	Sterol 27-hydroxylase
CYP7B1	Oxysterol 7α-hydroxylase
E2	17β-Estradiol
LBD	Ligand binding domain
NO	Nitric oxide
BMI	Body mass index
WAT	White adipose tissue
HFHC	High fat/high cholesterol
HER2	Human epidermal growth factor receptor 2
TNBC	Triple negative breast cancer
LTED	Long time estrogen deprivation
AR	Androgen receptor
LXR	Liver X receptor
